# Supplementary material for: Human-computer interaction based on background knowledge and emotion certainty
Source: PeerJ Comput Sci. 2023 May 31;9:e1418. doi: 10.7717/peerj-cs.1418 (PMC10280641; doi:10.7717/peerj-cs.1418)
Supplement: Supplemental Information 1 [file peerj-cs-09-1418-s001.zip › nlpcc2018.trainset/NLPCC-OpenDomainQA-2018.guideline.pdf]

NLPCC 2018 Shared Task Guideline:

Open Domain Question Answering

We call the *Open Domain Question Answering* shared task in NLPCC 2018, which includes the following three sub-tasks for **Chinese** language (the first two) and **English** language (the last one):

1) **Knowledge-Based Question Answering (KBQA)**
2) **Knowledge-Based Question Generation (KBQG)**
3) **Knowledge-Based Question Understanding (KBQU)**

For KBQA task, we will provide a train set and a test set. An example in train set is given below:

**<question id=”X”>**	你知道迪克牛仔是什么属相吗？
**<answer id=”X”>**	鼠

In train set, both questions and their golden answers will be provided. In test set, only questions will be provided. The participating teams should predict an answer for each question in test set. The format of the submission file should follow the same format above. If no answer can be predicted for a given question, just set the value of **<answer id=”X”>** to an empty string. The quality of a KBQA system will be evaluated by **answer exact match**.

For KBQG task, we will provide a train set and a test set. An example in train set is given below:

**<triple id=”X”>**	微软 ||| 创始人 ||| 比尔盖茨
**<question id=”X”>**	是谁创办了微软公司？

In train set, both triples and their golden questions will be provided. In test set, only triples will be provided. The participating teams should generate a natural language question for each triple in test set, and this generated question can be answered by the object entity of the given triple. The format of the submission file should follow the same format above. The quality of a KBQG system will be evaluated by **BLEU-4**.

For KBQU task, we will provide a train set and a test set. An example in train set is given below:

**<question id=”X”>**	what is fight songs of Maryland
**<logical form id=”X”>**	(lambda ?x (sports.team.fight_song Maryland ?x))

In train set, both questions and their golden logical forms will be provided. In test set, only questions will be provided. The participating teams should predicate a logical form for each question in test set. The format of the submission file should follow the same format above. The quality of a KBQU system will be evaluated by **logical form exact match**.
