# Supplementary material for: Human-computer interaction based on background knowledge and emotion certainty
Source: PeerJ Comput Sci. 2023 May 31;9:e1418. doi: 10.7717/peerj-cs.1418 (PMC10280641; doi:10.7717/peerj-cs.1418)
Supplement: Supplemental Information 3 [file peerj-cs-09-1418-s003.zip › ChatterBot_HCI_source_code/docs/_templates/layout.html]

{% extends '!layout.html' %}
{% set metatags = '' %}
{% block menu %}
{{ super() }}
{% endblock %}
