# Supplementary material for: Human-computer interaction based on background knowledge and emotion certainty
Source: PeerJ Comput Sci. 2023 May 31;9:e1418. doi: 10.7717/peerj-cs.1418 (PMC10280641; doi:10.7717/peerj-cs.1418)
Supplement: Supplemental Information 3 [file peerj-cs-09-1418-s003.zip › ChatterBot_HCI_source_code/examples/django_app/example_app/templates/nav.html]

{% load staticfiles %}

ChatterBot

- Documentation
- GitHub

- API
- Admin
