# Supplementary figures and images for: Human-computer interaction based on background knowledge and emotion certainty
Source: PeerJ Comput Sci. 2023 May 31;9:e1418. doi: 10.7717/peerj-cs.1418 (PMC10280641; doi:10.7717/peerj-cs.1418)

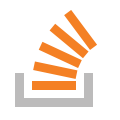

Supplement: Supplemental Information 3 [file peerj-cs-09-1418-s003.zip › ChatterBot_HCI_source_code/docs/_static/so-icon.png]

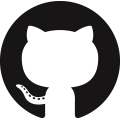

Supplement: Supplemental Information 3 [file peerj-cs-09-1418-s003.zip › ChatterBot_HCI_source_code/docs/_static/github-mark.png]

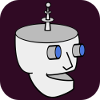

Supplement: Supplemental Information 3 [file peerj-cs-09-1418-s003.zip › ChatterBot_HCI_source_code/examples/django_app/example_app/static/img/chatterbot.png]
